# Efficacy of bladder instillations with adelmidrol and sodium hyaluronate for the treatment of symptomatic radiation cystitis

**DOI:** 10.3389/fsurg.2025.1735532

**Published:** 2026-01-27

**Authors:** Francesco M. Bracco, Enrico Ammirati, Alessandro Giammò, Paolo Gontero

**Affiliations:** 1 Urology Clinic, Department of Surgical Sciences, A.O.U. Città Della Salute e Della Scienza di Torino, University of Turin, Turin, Italy; 2 Neuro-Urology, CTO-Spinal Cord Unit, Città Della Salute e Della Scienza di Torino, Turin, Italy

**Keywords:** adelmidrol, bladder symptoms, hyaluronic acid, intravesical instillation, radiation cystitis, hematuria, pelvic pain

## Abstract

**Introduction:**

Radiation cystitis (RC) is a well–recognized complication of pelvic irradiation, manifesting as symptoms and signs such as hematuria, pain during urination, chronic pelvic pain, urgency and incontinence, which negatively impact patients' quality of life. We conducted a retrospective observational clinical investigation to evaluate the effect of bladder instillation with Adelmidrol (ADM) and sodium hyaluronate (HA) in patients with symptomatic RC.

**Methods:**

We collected data from the patients' clinical records for all patients treated in our hospital with a diagnosis of RC and undergoing a cycle of 8 weekly 60 min bladder instillations with 50 mL a solution of 2% ADM + 0.1% HA. We evaluated the presence of pain (measured with the VAS score), urinary urgency/frequency, macroscopic hematuria and incontinence (all registered as a dichotomous variable present/absent) before and at the end of the treatment.

**Results:**

Pelvic pain, evaluated by Visual Analogue Scale (VAS), improved in 66.7% of patients, significantly decreasing from a mean score of 3.8 ± 0.47–1.1 ± 0.3 (*p* < 0.0001). Gross hematuria and urgency, presented by 83.3% and 90.0% of patients, were reported by 6.7% and 33.3%, respectively, after the end of treatment (*p* < 0.0001). Incontinence, at first reported by 43.3% of the considered patients, disappeared in 16.7% of the subjects (n.s.).

**Discussion:**

These results suggest the beneficial effect of ADM + HA intravesical instillations in managing RC symptoms, especially pain, gross hematuria and urgency.

## 1 Introduction

Radiotherapy (RT) is a widely used treatment for many pelvic malignancies, including gastrointestinal, gynecological and urological tumors (1, 2), but it may expose pelvic organs to the risk of radiation-induced injuries (3). While RT targets the tumor with high-energy radiation, the bladder, urethra, and distal ureters also receive some of the radiation dose. Over the years, the improvement of RT techniques has allowed the administration of increasingly effective doses in smaller volumes with a noticeable amelioration in treatment tolerance (4, 5). The bladder remains an extremely sensitive organ even to low doses of radiation, which are responsible for the subsequent acute and late adverse events (6, 7). The response of the bladder to RT, in fact, can be classified into acute/subacute reactions, occurring within three to six months of radiation treatment, and late side effects, appearing after six months (3).

Radiation cystitis (RC), defined as the manifestation of symptoms and signs such as hematuria, pain during urination, increased urinary frequency, urgency, and incontinence (8, 9). Its general incidence ranges from 9.1% to 80%, with a wide variability due to the methods and doses of radiotherapy used among different medical subspecialties. The acute symptomatic type is estimated to have an incidence of nearly 50% following pelvic irradiation at full curative doses, while the incidence of late RC is approximately 5%–10% (5). The condition typically develops around 31.8 months following treatment, with males more affected than females (2.8:1) likely due to the frequent use of radiotherapy in the treatment of prostate cancer (3). Additionally, the actual risk of developing hematuria, the main presenting symptoms of radiation cystitis, is 5.8% at five years and 9.6% at twenty years (3). Early symptoms arise from the disruption of tight junctions and the glycosaminoglycan (GAG) layer, which leads to increased urothelial permeability and allows urine and tissue to come into contact, causing irritability and an inflammatory response of the bladder wall (10, 11). Because the bladder epithelium has a low proliferative rate, urothelial cells show signs of damage within three months following radiation, with an increased endothelial cell proliferation and perivascular fibrosis after 6–12 months (6). As a result, late radiation tissue injury develops over months to years. Symptoms during the late-response phase may result from vascular injury leading to focal bladder ischemia (6, 10), which promotes edema, cellular destruction and bladder smooth muscle fibers substitution with fibroblasts (12).

Despite the availability of several therapeutic options, such as astringent agent instillations, hyperbaric oxygen therapy, or cystectomy in the most severe cases, treatments for RC are often time-consuming and may lead to side effects, without reversing bladder damage. Additionally, many patients may experience relapses, highlighting the huge need to develop new strategies for managing the condition (13).

Adelmidrol (ADM), a diethanolamide derivative of azelaic acid analogue of palmytoylethanolamide (PEA) (14), in association with hyaluronic acid (HA), has already shown to be an effective treatment option for conditions associated with urothelial damage (15), confirmed by clinical studies on interstitial cystitis/painful bladder syndrome (IC/BPS) (15) and on patients undergoing intravesical antitumor therapy after surgery for non-muscle invasive bladder cancer (16, 17).

ADM acts as a preventive antioxidant and counteracts the production of reactive oxygen (ROS) and nitrogen (RNS) species, that are frequently produced after RT; ADM also prevents HA oxidative degradation, a key component of the GAG layer (18–22) ADM has an indirect anti-inflammatory effect, preventing the activation of mast cells (MC), which can release vasoactive, nociceptive and proinflammatory mediators (including TGF-ß) leading to neuronal sensitization and secretion of other neurotransmitters and neuropeptides, which in turn can further stimulate MCs activation (23–26), and switch the local and acute inflammatory response into a chronic and systemic inflammatory disease, with amplification of painful stimuli too (27).

Considering all this evidence, we decided to retrospectively collect data from patients who developed symptomatic RC and were treated with ADM + HA bladder instillations.

## 2 Materials and methods

### 2.1 Study design and participants

This retrospective observational study evaluated the effect of ADM + HA bladder instillations in patients who experienced symptomatic RC following radiotherapy for different types of cancer (i.e., prostate, bladder, hematological or gynecological tumor). We collected data from patients afferent to the Molinette University Hospital of Turin (Italy) between February 2020 and March 2023, who complained the following symptoms for at least 3–6 months: increased urinary frequency (<2 h), objective macroscopic hematuria (associated or not with clots), urinary urgency with or without incontinence episodes (use of 1 or more pads/day), pelvic pain. Patients undergoing chemotherapy, who received another treatment for RC the month before ADM + HA instillations, with urethral stricture disease and lower urinary tract infections were excluded from the study.

Each patient underwent 8 bladder instillations (once a week) with a solution composed of 1,000 mg (2%) of ADM and 50 mg (0.1%) of HA (Vessilen®, Epitech Group Spa, Saccolongo, Padova), delaying urination for at least 60 min after instillation. All patients with persistence of symptoms (pain, urgency or haematuria) were offered the possibility to plan a second instillation cycle.

The data utilized in the research were derived exclusively from historical clinical records. To safeguard the privacy of the patients, all personal identifiers, such as names, dates of birth, or any information that could potentially reveal the identity of individuals, were meticulously removed during the data preparation process. This ensured that the data were fully anonymized and untraceable. Furthermore, the patients whose data were included had previously provided explicit consent after receiving clear and comprehensive information about the nature of the research. The study was conducted in accordance with Helsinki Declaration and the principles of Good Clinical Practice (GCP).

### 2.2 Assessments

All evaluations were performed before starting treatment with ADM + HA, and after the last intravesical instillation, according to the clinical practice in force in our clinic.

Pelvic pain was assessed using Visual Analogue Scale (VAS), a tool that evaluates the intensity of pain symptoms perceived by the patient. It is represented by a line, 10 cm long, where one end (0) indicates the absence of the symptom, and the other end (10) represents the worst imaginable symptom intensity.

Hematuria, urgency and incontinence were evaluated by questioning the patient about the presence/absence of the symptoms before and after the treatment period.

### 2.3 Statistical analysis

Statistical analysis was conducted using the Signed Rank Test. All values are expressed as mean ± standard deviation (SD) or standard error of the mean (SEM), as specified. *P*-values less than 0.05 were considered as statistically significant. Data were analyzed using SAS v9.4 (SAS Institute, Carry, NC, USA).

## 3 Results

### 3.1 Patients characteristics

A total of 30 patients (10 females and 20 males) with a mean age of 68 ± 18.25 years, were included in the study. Each patient received different total radiation doses based on treatment modality, cancer type, and tumor location. Fourteen patients were treated with RT for prostate cancer, seven for hematological tumor, six for gynecological cancer including cervix, breast, and ovary tumor, two for bladder cancer, and one for sarcoma (Table 1).

**Table 1:** Patients’ cancer type and symptoms.

Tumor, *n* (%)	Symptoms, *n* (%)			
	Hematuria 25 (83.3)	Urgency 27 (90.0)	Incontinence 13 (43.3)	Pelvic pain 20 (66.7)
Prostate, 14 (46.7)	14 (100)	12 (85.7)	11 (78.6)	8 (57.1)
Hematological, 7 (23.3)	7 (100)	7 (100)	0	4 (57.1)
Gynecological, 6 (20.0)	2 (33.3)	5 (83.3)	1 (16.7)	5 (83.3)
Bladder, 2 (6.7)	1 (50)	2 (100)	0	2 (100)
Sarcoma, 1 (3.3)	1 (100)	1 (100)	1 (100)	1 (100)

*n*, number of patients.

All patients complained of two or more symptoms before starting treatment with ADM + HA intravesical instillation. Depending on the type of cancer, patients experienced hematuria, urgency, incontinence and pelvic pain (VAS≥3) with a different prevalence, as reported in Table 1.

Only two patients performed more than the 8 planned ADM + HA instillations, due to symptoms persistence. Specifically, one patient received a total of 11 instillations and another received 14.

### 3.2 Evaluation of RC symptoms

#### 3.2.1 Reduction of pain

**Figure 1:**
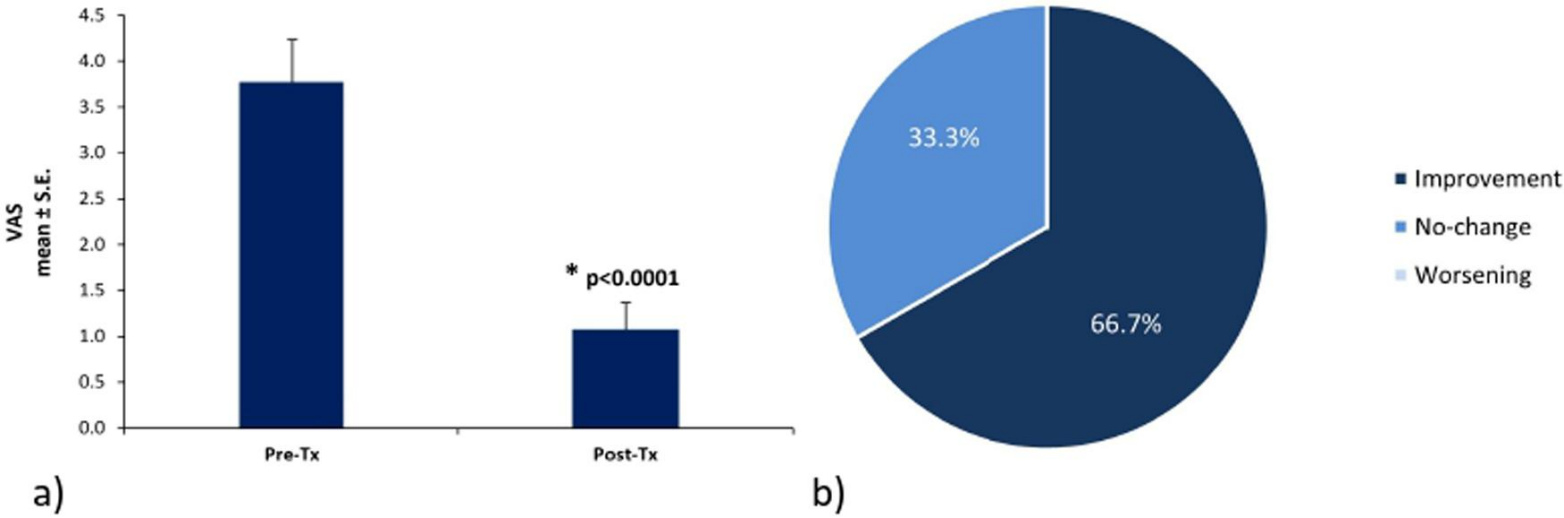
**(a)** Pain intensity before and after ADM + HA bladder instillations, *significant change in pain intensity between pre- and post-treatment (signed rank test); **(b)** percentage of patients reporting improvement, no-change or worsening of pelvic pain. Tx, treatment.

Pain severity decreased from a mean VAS score of 3.8 ± 0.47 before ADM + HA treatment to 1.1 ± 0.30 at the end of the observation period (*p* < 0.0001) (Figure 1a). A reduction in pain intensity of 2 or more points on the VAS scale, considered a clinically significant improvement, was reported by 20 patients (66.7%). 10 patients (33.3%) indicated that their pain remained unchanged; consequently, none worsened. Among the 10 patients with no change, three had no pain from the beginning and seven showed an improvement <2 points on the VAS scale (Figure 1b).

#### 3.2.2 Presence and absence of hematuria, urgency, and incontinence

**Figure 2:**
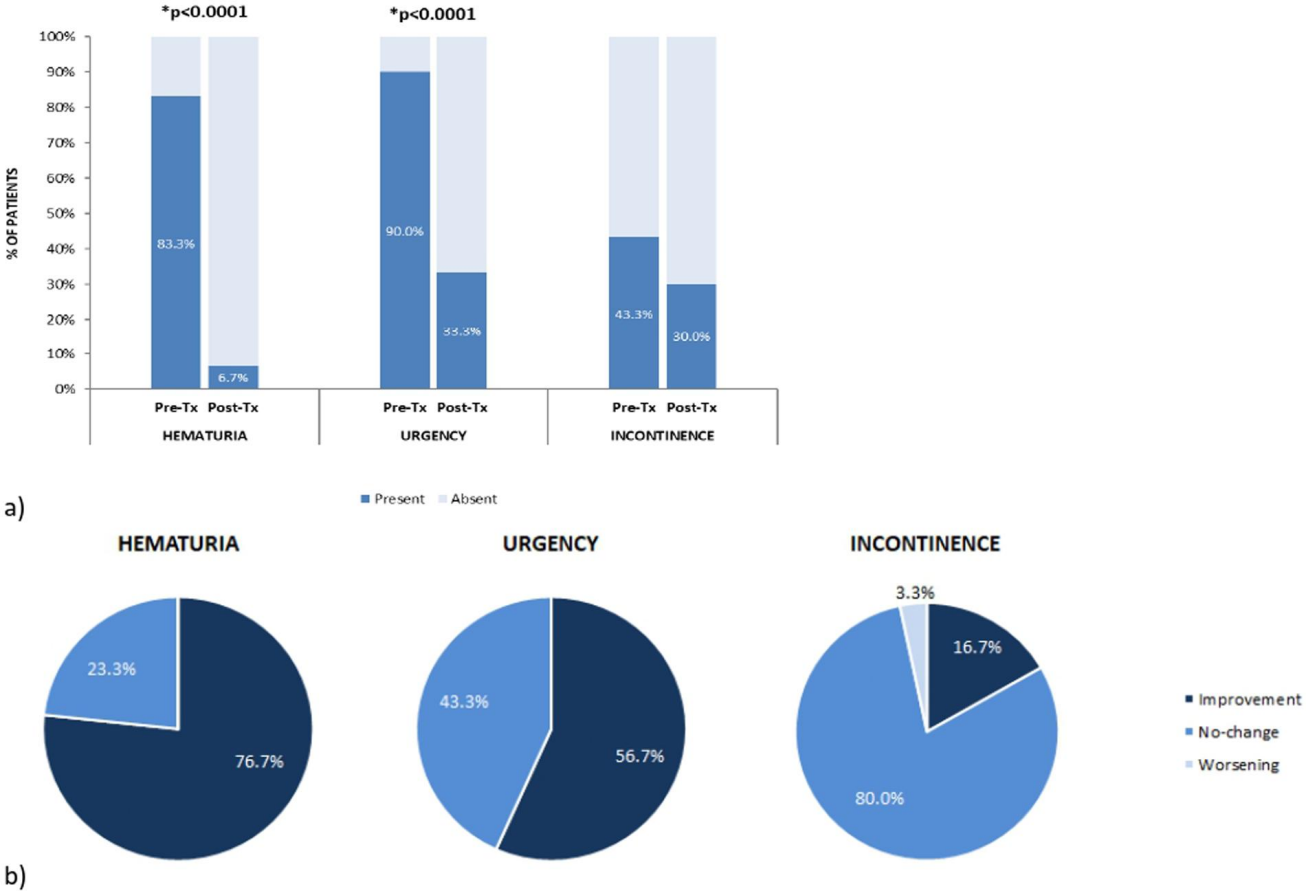
**(a)** Hematuria, urgency and incontinence distribution among patients before and after ADM + HA bladder instillations. Dark blue values indicate the presence of the symptom before and after treatment. **(b)** Percentage of patients reporting symptoms improvement (presence of the symptom before treatment and absence after), no-change (absence or presence of the symptom both before and after treatment) or worsening (absence before treatment and presence at the end of the observational period). *Significant symptom presence/absence variation between pre- and post-treatment (Signed Rank Test); Tx, treatment.

Gross hematuria, reported by 25 out of 30 patients (83.3%) before ADM + HA treatment, was present in only two patients (6.7%) after the last intravesical instillation, resulting in a symptom resolution of 92%. Considering all the 30 patients, 23 (76.7%) showed an improvement of this condition, reporting that it was no longer present at the end of the treatment; seven (23.3%) declared no change in its presence/absence (five of them had no hematuria at the beginning of treatment, two presented the symptom both at the beginning and at the end of treatment); none showed worsening of the symptom, defined as hematuria appearance during treatment. Overall, a significant variation in the presence of gross hematuria was observed from the beginning to the end of the observational period (*p* < 0.0001) (Figures 2a,b).

Similarly, urgency was present in 27 patients (90.0%) at the pre-treatment time point, and in only 10 patients (33.3%) at the end of treatment, decreasing by 63.0%. Of all patients considered, urgency disappeared in 17 (56.7%) and remained unchanged in 13 (43.3%). Among the latter, three didn't have the symptom at the beginning of the treatment. No one showed worsening, thus resulting in a significant variation in the number of patients presenting urgency before and after ADM + HA instillations (*p* < 0.0001) (Figures 2a,b). Two patents with persistence of symptoms of urgency and gross haematuria, accepted a second cycle of HA + ADM instillations, with complete resolution of both urgency and gross haematuria.

Finally, 13 patients (43.3%) suffered from incontinence before starting bladder instillations and 9 (30.0%) presented it at the post-treatment check-up, indicating a variation in the presence of the symptom of 30.7%. In most cases, i.e., in 24 out of 30 patients (80%), symptom presence/absence remained unchanged (six of these patients did not have incontinence even at the beginning of the treatment). Incontinence disappeared in five patients (16.7%) and worsened in only one (3.3%). Overall, no significant variation in the presence/absence of the symptom was observed at the post-treatment evaluation compared to pre-treatment (n.s.) (Figures 2a,b).

We confirmed the persistence of clinical benefit in patients who responded to treatment six months after the end of the bladder instillations. No adverse effects were reported during the entire course of the ADM + HA intravesical instillations.

## 4 Discussion

The integrity of bladder urothelium is indispensable for the health of this organ. It is well known that urothelial damage compromises the function of the blood-urine barrier, leading to urinary diseases and macroscopic hematuria (28, 29). Besides forming a barrier, the urothelium plays also an important role in the sensory function of the bladder (30). Chronic pathological conditions can, in fact, alter the sensory pathways, leading to a reduction in pain threshold and/or to an amplification of painful perception. This increased sensation can result from changes in the properties, density, and/or stimulation threshold of peripheral nociceptive afferents or from changes within the central pathways that process nociceptive inputs (31). Concurrently, when the GAG layer is damaged, potassium penetrates into the bladder wall causing the activation of C-fibers which promote smooth muscle contraction, neurogenic inflammation, and hypersensitivity. Therefore, restoration of the GAG layer and reduction of mast cell-mediated inflammation may represent the goal of radiation cystitis treatment (32). To date, in fact, RC remains challenging to manage due to its complex and only partially understood pathophysiology. An alternative approach for patients with this condition can be represented by the instillation of HA, given the diverse mechanisms of action of this molecule and its already known efficacy in treating interstitial cystitis (33). Evidence regarding the effectiveness and optimal regimen of hyaluronic acid instillations as a therapy for RC, however, is limited due to the small number of clinical trials and lack of consistent data (34, 35). Despite that, the few studies performed have shown positive outcomes, evidenced by improvements in patients' symptoms, such as recovery from voiding dysfunction, reduced urinary frequency, hematuria and pelvic pain, as well as enhanced bladder capacity and quality of life (32, 36–39) Anyhow, as previously mentioned, ionizing radiations induce the production of ROS and RNS responsible for oxidative stress, which causes urothelial damage, inflammation and fibrosis resulting in urinary symptoms (20, 21), and also for HA degradation, occurring at higher levels during tissue injury and inflammatory processes (40).

ADM, by counteracting oxidative stress thanks to its hydroxyl radical scavenging activity, is able to preserve HA from oxidative degradation (18). As a consequence, ADM + HA intravesical instillation may offer a promising unique advantageous and effective treatment that can facilitate the repair of the GAG layer, significantly alleviating symptoms like pain and urgency. The results of the present retrospective investigation, conducted on patients with symptomatic RC associated with gross hematuria, urinary urgency, incontinence episodes, and pelvic pain, highlighted a significant reduction in the symptoms experienced by patients, in particular macroscopic hematuria and urgency, after the end of treatment. Despite fewer patients suffered from incontinence at the end of treatment, no significant variation in the presence/absence of the symptom was observed, probably because almost half of the patients had undergone pelvic surgery (radical prostatectomy). This may have had an impact on the number of patients experiencing the symptom and, consequently, on its subsequent improvement. The significant reduction in pelvic pain is instead in agreement with a previous study conducted on patients with IC/BPS, in which intravesical ADM + HA significantly ameliorated both the intensity of the symptom and the quality of life (15).

In addition, ADM, by preventing the generation of small molecules induced by radicals, can mitigate mast cells hyperactivation and the cascade of events that follow. In response to stimuli, in fact, bladder MCs release numerous inflammatory mediators, which thus provide these cells a central contribution in the inflammatory processes underlying bladder pathologies. This role is supported by the clinical evidence of an increased number of MCs in several bladder syndromes (e.g., bladder cancer, interstitial and chronic cystitis) (41), which may be related to injury and/or alterations of the urothelial cells, resulting in cytokines and nerve growth factor (NGF) production, which, in turn, can further stimulate MCs proliferation and/or activation (42). NGF, in particular, is a well-known contributor to urinary bladder dysfunction because, besides mediating inflammation, is involved in the morphological and functional changes which can occur in sensory and sympathetic neurons innervating the bladder (43, 44). At the same time, this mediator has also been implicated in the development of hyperalgesia by acting directly on sensory nerve endings or indirectly by increasing the expression of neuropeptides, such as substance P, which in turn can upregulate NGF expression and mediate some of the peripheral and central effects of inflammation (31). Being MCs commonly found in the proximity of SP-positive fibers, it is suggested that upon their degranulation, the factors released can easily target afferent nerves, urothelial cells and other structures in the bladder, contributing to its pathology (45).

The treatment of RC with only HA has been previously evaluated, although the main outcome differ between studies, making a direct comparison difficult. Shao et al. evaluated the results of intravescical administration of HA for RC and compared them with hyperbaric oxygen; 16 patients were enrolled in the HA arm and they experienced a decrease in voiding frequency up to 12 months (−1.5 ± 1.4 voids/day) and a reduction of the VAS score (−1.31 ± 1.3), without mentioning the recurrence of haematuria; the authors reported an augmented risk of urinary tract infections (42.8% vs. 10%, *P* = 0.034) at 6 months, not confirmed at 12 and 18 months (37). Gacci et al. reported the results of the instillation of HA + chondroitin sulfate in a group of 30 patients with significant LUTS after prostate RT. After a cycle of intravescical instillations, the authors reported a reduction in symptom score of the ICSI/ICPI questionnaire (*P* < .001 and *P* = .006), and a restoration to baseline values of the symptoms and bother of urgency, frequency, nocturia and pain; even in this case there is no mention about haematuria (46). Sanguedolce et al. evaluated the results of HA + chondroitin sulfate for the treatment of RC in a population of 51 patients after pelvic RT; among them 35 patients required hospitalization, 22 transurethral fulguration and 10 a second intravescical instillation cycle. After a median follow-up of 59 months 36/51 patients had none significant haematuria episodes (47).

The findings of this study thus indicate that ADM + HA bladder instillations may represent an effective treatment option for optimizing the restoration of the urothelial coating compromised by inflammation and oxidative stress. However, despite the promising results, the study has some limitations. The retrospective nature of the study, without a control group, does not rule out the possibility of patient selection bias, as the results were analyzed by searching data from previous electronic records. The impossibility of prospectively evaluating patients made it necessary to categorize symptoms as dichothymic variables, preventing the assessment of urinary frequency, number of urgency episodes and the results of validated questionnaires for all patients. The sample size is reasonable, but certainly not large enough to draw definitive conclusions. Further studies with a perspective-controlled setting, with longer follow-ups and more patients included, are thus needed to deeply investigate the beneficial effects of ADM + HA in radiation-induced cystitis.

## 5 Conclusions

This study demonstrated significant reductions in gross hematuria, urgency, and pelvic pain in patients with chronic radiotherapy-induced cystitis. Therefore, intravesical instillations of ADM in combination with HA appear to be an effective and safe treatment option.

## Data Availability

The raw data supporting the conclusions of this article will be made available by the authors, without undue reservation.
