# Supplementary material for: Efficacy of bladder instillations with adelmidrol and sodium hyaluronate for the treatment of symptomatic radiation cystitis
Source: Front Surg. 2026 Jan 27;12:1735532. doi: 10.3389/fsurg.2025.1735532 (PMC12888785; doi:10.3389/fsurg.2025.1735532)
Supplement: Supplementary file 1 [file Table1.docx]

| **Patient** | **Age** | **Cause of RT** | **Hematuria** | **Urgency** | **Incontinence** | **Pelvic pain** |
| --- | --- | --- | --- | --- | --- | --- |
|  |  |  | 0=absent 1= present | | | VAS (0-10) |
| 1 | 62 | Acute Myeloid Leukemia | 1 | 1 | 0 | 0 |
| 2 | 61 | Acute Myeloid Leukemia | 1 | 1 | 0 | 3 |
| 3 | 44 | Acute Myeloid Leukemia | 1 | 1 | 0 | 8 |
| 4 | 23 | Acute Myeloid Leukemia | 1 | 1 | 0 | 7 |
| 5 | 58 | Acute Myeloid Leukemia | 1 | 1 | 0 | 0 |
| 6 | 45 | Acute Myeloid Leukemia | 1 | 1 | 0 | 5 |
| 7 | 27 | Hodgkin linfoma | 1 | 1 | 0 | 1 |
| 8 | 51 | Pelvic sarcoma | 1 | 1 | 1 | 8 |
| 9 | 68 | Gynecological cancer | 0 | 1 | 1 | 8 |
| 10 | 71 | Gynecological cancer | 1 | 1 | 0 | 0 |
| 11 | 64 | Gynecological cancer | 0 | 1 | 0 | 6 |
| 12 | 68 | Gynecological cancer | 0 | 1 | 0 | 8 |
| 13 | 73 | Gynecological cancer | 1 | 0 | 0 | 3 |
| 14 | 77 | Gynecological cancer | 0 | 1 | 0 | 3 |
| 15 | 76 | Bladder cancer | 0 | 1 | 0 | 8 |
| 16 | 90 | Bladder cancer | 1 | 1 | 0 | 4 |
| 17 | 81 | Prostate cancer | 1 | 1 | 1 | 6 |
| 18 | 75 | Prostate cancer | 1 | 1 | 1 | 3 |
| 19 | 93 | Prostate cancer | 1 | 1 | 1 | 3 |
| 20 | 66 | Prostate cancer | 1 | 1 | 1 | 2 |
| 21 | 88 | Prostate cancer | 1 | 1 | 1 | 2 |
| 22 | 72 | Prostate cancer | 1 | 1 | 1 | 1 |
| 23 | 98 | Prostate cancer | 1 | 1 | 1 | 3 |
| 24 | 85 | Prostate cancer | 1 | 1 | 1 | 3 |
| 25 | 81 | Prostate cancer | 1 | 0 | 1 | 2 |
| 26 | 87 | Prostate cancer | 1 | 1 | 1 | 3 |
| 27 | 78 | Prostate cancer | 1 | 1 | 1 | 2 |
| 28 | 90 | Prostate cancer | 1 | 0 | 0 | 5 |
| 29 | 86 | Prostate cancer | 1 | 1 | 0 | 2 |
| 30 | 75 | Prostate cancer | 1 | 1 | 0 | 4 |

Supplementary table 1: baseline patients’ characteristics

Legend RT= radiotherapy
